# Atypical glandular cells and development of cervical cancer: Population‐based cohort study

**DOI:** 10.1002/ijc.34242

**Published:** 2022-08-27

**Authors:** Ingrid Norman, Emel Yilmaz, Anders Hjerpe, Maria Hortlund, Klara Miriam Elfström, Joakim Dillner

**Affiliations:** ^1^ Department of Laboratory Medicine Karolinska Institutet Stockholm Sweden; ^2^ Department of Clinical Pathology and Cancer Diagnostics, Center for Cervical Cancer Prevention, Medical Diagnostics Karolinska Karolinska University Hospital Stockholm Sweden; ^3^ LINK Medical Research AB Malmö Sweden

**Keywords:** atypical glandular cells, cervical intraepithelial, HPV genotyping

## Abstract

The effect of cervical screening on cervical adenocarcinoma has been variable, possibly because the risk associated with the precursor atypical glandular cells (AGC) is not well known. A cohort of all 885 women in the capital region of Sweden with AGC, a concomitant human papillomavirus (HPV) analysis, and a histopathology was followed until 2019. Cumulative incidence proportions of cervical intraepithelial lesion grade 3 or worse (CIN3+) by HPV type was determined by 1‐Kaplan‐Meier estimates. Hazard ratios (HR) for CIN3+ or for invasive cancer were estimated with Cox regression. After 2 years of follow‐up, the cumulative incidence proportions of CIN3+ were 80% (95% confidence interval [CI]: 74‐86%), 58% (95% CI: 50‐60%) and 10% (95% CI: 5‐18%) among HPV16/18 positive, “other HPV” positive and HPV‐negative women, respectively. Among the 300 women with HPV16/18 positive AGC, 217 developed CIN3+ of which 35 were invasive cervical cancer. The 2‐year cumulative invasive cancer risk for HPV16/18 positive AGC was 17% (95% CI: 12‐24%). Primary HPV‐screening had a similar yield of CIN3+ as cytology screening, albeit HPV‐negative AGC is by design not detected by HPV screening. Among 241 women with HPV‐negative AGC, 11 developed CIN3+ mostly after clinically indicated samples. We found no significant risk differences depending on age or sampling indication. The low CIN3+ risk after HPV‐negative AGC implies safety of primary HPV screening. The high risk of invasive cervical cancer after HPV16/18 positive AGC implies that management of this finding is a priority in cervical screening.

## Graphical abstract

What's new?

The impact of cervical screening on cervical adenocarcinoma prevention has been variable, possibly because the cancer risk associated with the precursor atypical glandular cells (AGC) is not well known. Here, the authors show that atypical glandular cells (AGC) positive for high‐risk human papilloma virus (HPV)16/18 confer very high risk of invasive cervical cancer (17%), whereas HPV‐negative AGC confer very little risk. The low invasive cancer risk after HPV‐negative AGC testing provides reassurance that HPV‐based primary screening is safe. The high cancer risk after HPV16/18‐positive AGC testing warrants a more ambitious and urgent management of women with this lesion.

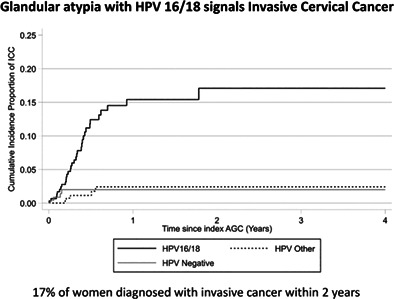

**Abbreviations**
ADCAcx: cervical adenocarcinoma
AGC: atypical glandular cells
AIS: cervical adenocarcinoma in situ
ASCUS: atypical squamous cells of undetermined significance
CI: confidence interval
CIN3+: cervical intraepithelial lesion grade 3 or worse
ERB: ethical review board
HPV: human papillomavirus
HR: hazard ratio
HSIL: high‐grade squamous intraepithelial lesion
ICC: invasive cervical cancer
LBC: liquid‐based cytology
LSIL: low‐grade squamous intraepithelial lesions
NKCx: the Swedish national cervical screening registry
RHS: randomized healthcare study
SCC: cervical squamous cell carcinoma
SCCA: squamous cell cancer
SNOMED: systematized nomenclature of medicine

## 1 INTRODUCTION

Cervical screening with cytology has been effective in reducing the incidence of cervical squamous cell carcinoma (SCC). However, the effect has been smaller and variable regarding prevention of cervical adenocarcinoma (ADCAcx). Indeed, many countries have even experienced an incidence increase in cervical adenocarcinoma in situ (AIS) and ADCAcx, in particular among women under the age of 50.^1^ In screened populations, ADCAcx typically accounts for 20% or more of all cervical cancer.^2^

ADCAcx is commonly not diagnosed until invasive cancer has already developed, resulting in poor prognosis.^3^ Atypical glandular cells (AGC) are precursors to ADCAcx^4^ but may also mark high‐grade squamous intraepithelial lesion (HSIL) and squamous cell cancer (SCCA). Nationwide registry linkage studies have found that management of AGC is variable and that there is a high and persistent risk of cervical cancer for many years after AGC in cytology.^5^

The proportion of cervical samples with AGC in cytology may vary from 0.18% to 0.74%.^6^ AGC is commonly regarded as a mild change, but the high cancer risk rather points to that intense follow‐up is needed.^7^

Different types of HPV have different cancer risks, with HPV16 and 18 having the highest risks. While HPV16 can cause all histological types of cervical cancer, HPV18 is particularly associated with ADCAcx.^8^, ^9^

AGC is of paramount importance for cervical screening strategies for several reasons: Most of the improved cancer‐protective effect seen with HPV‐based screening was seen for prevention of ADCAcx^10^ and the only reasonable explanation for this finding is that a better identification of ADCAcx precursors was enabled. Management of AGC may differ in different settings as AGC may either reflect high‐risk precancerous lesions or mere reactive changes.^6^ Strategies with HPV‐based triaging of AGC have not been widely implemented, possibly because the evidence base on the predictive values of HPV testing in AGC is limited. However, HPV‐based primary cervical screening is being widely rolled out and HPV‐negative AGC will thus never be detected or followed up in the modern‐day HPV‐based primary screening strategy. A solid evidence base on the progressive potential of AGC in relation to HPV status is therefore essential.

We sought to provide a solid evidence base on the progressive potential of AGC in a setting suitable for this. The organized screening program in the capital region in Sweden decided on February 17, 2014 to introduce reflex HPV testing for all women with AGC, while retaining the same management guidelines for both HPV‐positive and HPV‐negative women with AGC. At the same date, the central laboratory introduced reflex HPV testing of all samples diagnosed with AGC (also when found in clinical testing). We had previously evaluated HPV reflex testing of atypical squamous cells of undetermined significance (ASCUS) and low‐grade squamous intraepithelial lesions (LSIL) in a randomized manner.^11^ However, as AGC is rather uncommon, we decided that a population‐based cohort study enrolling all women with AGC over several years would be preferable to provide evidence on the progressive potential of AGC in relation to HPV status.

## 2 MATERIALS AND METHODS

### 2.1 Study population and data collection

The cervical screening program in Sweden targets all women ages 23 to 70. At the time of the study, 3‐year intervals were used between ages 23 to 50 and 5‐year intervals were used over the age of 50. Samples can also be taken outside of the program if a physician decides that there is a clinical indication to do so. In the region of Stockholm, HPV‐based screening was piloted in a randomized healthcare study (RHS) between 2014 and 2016, where all resident women ages 30 and above were randomized to either HPV‐based screening or cytology‐based screening.^12^ HPV‐based screening was implemented as the primary analysis for all resident women ages 30 and above on January 1, 2017. For the age group 23 to 29 years, all women were offered primary cytology screening also during the time when the RHS was performed. After the end of the RHS on January 1, 2017, women were offered primary cytology screening in the ages 23 to 29 and primary HPV screening in the ages 30 to 70 years.

The choice of test for clinically indicated samples was mostly primary cytology with reflex HPV testing in the early years, but co‐testing was increasingly used in recent years.

The regional screening registry collects data on all cytologies and cervical histopathologies in the region, also from outside the program. All regional registries in the country send in their data to the Swedish national cervical screening registry (NKCx).^13^

The population‐based cohort in this study was defined as all women resident in the capital region of Sweden who had had a cervical sample with an AGC diagnosis between February 17, 2014 and December 31, 2018 (regardless of whether the sample was taken in the screening program or because of clinical testing). Adenocarcinoma in situ (AIS) is not included in the AGC diagnoses. Sub‐diagnoses of AGC (AGC no other specification and AGC favor neoplasia) are not used in Sweden. Comparison with the NKCx identified if a corresponding HPV test (result within 40 days of the cytology) had been done and if there was a subsequent histopathological follow‐up. As per national guidelines, all women with an AGC should be referred for colposcopy‐directed biopsy with histopathological assessment. The registry follow‐up of the study ended on December 31, 2019.

All cervical cytology samples used the liquid based cytology, LBC (ThinPrep) technology. The samples were taken with plastic spatula and endocervical brush (Medscand; Cooper Surgical Company, Berlin, Germany). The cells were collected from the ectocervix and endocervix of the uterus and suspended in PreservCyt, a methanol‐based fixative medium, according to the manufacturer's recommendation (ThinPrep; Hologic, Marlborough, Massachusetts). LBC samples were transferred to cytology glass slides using ThinPrep processors (Hologic), before Pap staining. The cell suspension was used for HPV DNA analysis using the Cobas 4800 HPV test (Roche), with decapping of ThinPrep vials (p480; Roche Molecular Diagnostic, Pleasanton, California). HPV types detected using Cobas included HPV 16, 18, 31, 33, 35, 39, 45, 51, 52, 56, 58, 59, 66 and 68, where HPV16 and HPV18 were detected in separate channels and the remaining HPV types (“other”) were detected in a pooled group. All LBC samples were prepared and evaluated at the Department of Pathology & Cancer Diagnostics, Medical Diagnostics Karolinska, Stockholm, Sweden. Cytological diagnoses were classified according to the Systematized Nomenclature of Medicine (SNOMED) code system. The Bethesda nomenclature was used, except that there was no subgrouping within the AGC diagnosis.^14^

### 2.2 Statistical analysis

The main outcome of interest was cervical intraepithelial neoplasia grade 3 or worse (CIN3+), including adenocarcinoma in situ and invasive cervical cancer. The risk for CIN3+ was evaluated by HPV type, hierarchically classified as HPV16 positive, HPV18 positive (not 16 positive), “other HPV” positive (not 16 or 18 positive), or HPV negative. Cumulative incidence proportions of CIN3+ by HPV status were calculated using 1‐Kaplan‐Meier (KM) estimate. Follow‐up time was calculated from the date of the index AGC sample to the first diagnosis of CIN3+ or the date of the last histopathological sample taken during study follow‐up if no CIN3+ was detected.

To estimate the relative risk for CIN3+ by HPV type among women with AGC; hazard ratios (HR) were calculated using Cox proportional hazards regression model. Unadjusted HRs with corresponding 95% confidence intervals (CI) were calculated for HPV‐positive AGC (16, 18, or “other HPV” positive) as well as for age in 5‐year categories with women under 30 as a reference for the models including women with a primary cytology sample or a clinically indicated sample. Age 30 to 34 was used as the reference for the models including women with a primary HPV sample given that this analysis method was recommended from age 30 and above (very few observations for primary HPV tests before 30).

All analyses were stratified by the primary analysis of the index sample, accounting for the period of the RHS by splitting the organized cytology and HPV samples into randomized and nonrandomized.

Data preparation was performed in SAS 9.4 (Cary, NC) and the analysis was performed in STATA 16 (College Station, TX).

## 3 RESULTS

**FIGURE 1:**
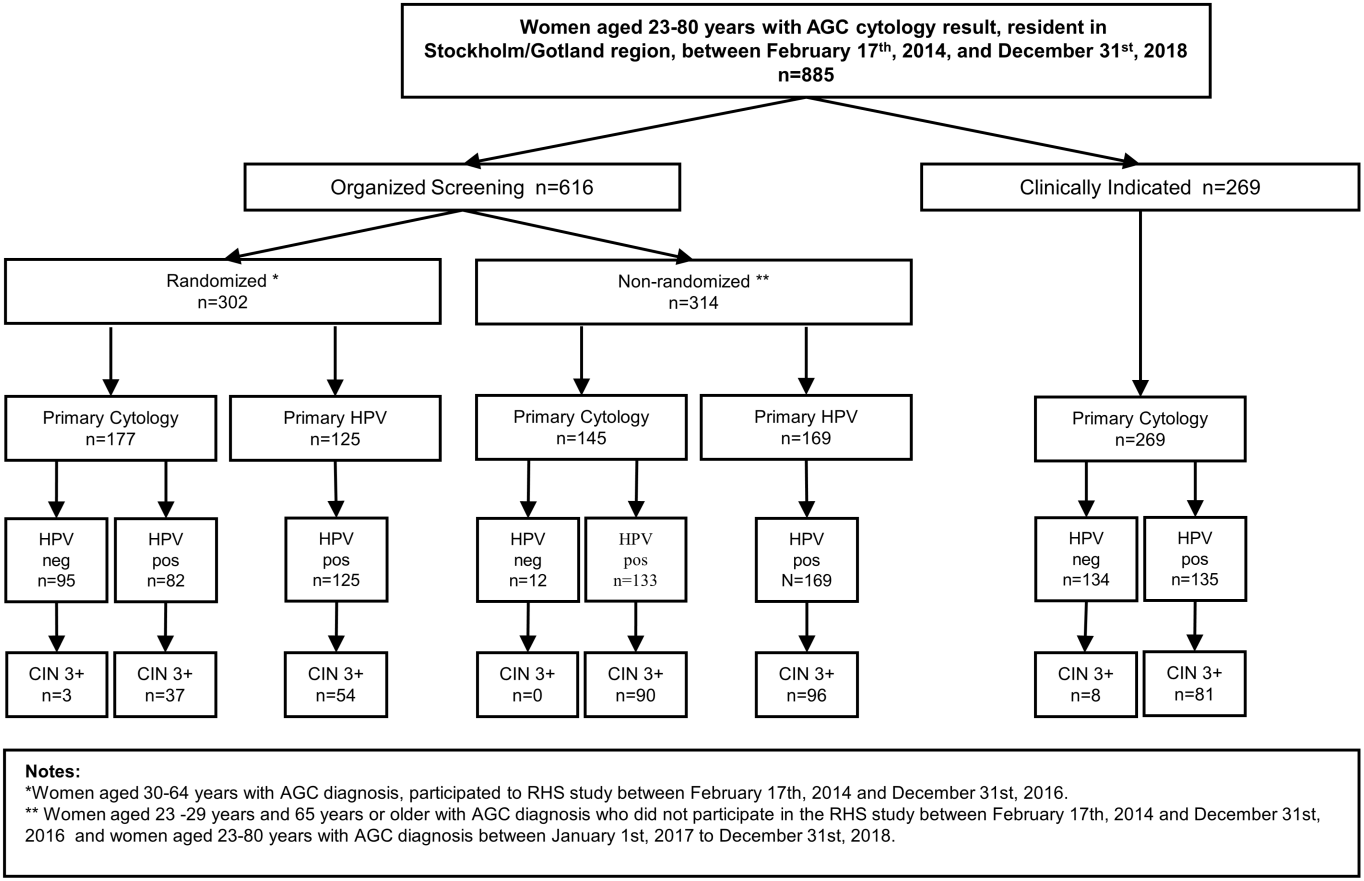
The study flow chart. All organized and nonorganized samples, HPV tests and histopathologies in the country were registered and followed. Nonorganized samples are supposed to be taken on a clinical indication for taking a cervical sample, but the exact reason is not registered. During 2014 to 2016, the organized screening program in the capital region of Sweden randomized all resident women to either being invited to primary cytology or to primary HPV. Since 2017 all women in the ages 30 to 70 are invited to primary HPV screening and women aged 23 to 29 to primary cytology screening (nonrandomized). All histopathologies after the AGC were recorded and the endpoint reached if a CIN3+ lesion had been diagnosed

A total of 885 women with an AGC diagnosis between February 17, 2014 and December 31, 2018 were identified. There were 616 women who were screened by the organized program and 269 women who had an AGC in a sample from clinical testing. In total, 369 CIN3+ cases were identified during follow‐up (325 CIN3/AIS, and 44 invasive cervical cancer [ICC] cases) of which 11 CIN3+ cases were among women with an HPV‐negative index AGC sample (three in women with an organized screening sample and eight among women with a clinically indicated sample) (Figure 1). Among the ICC cases, 33 were adenocarcinomas, nine were squamous cell carcinomas, and two were other, rare cervical cancer types.

In the follow‐up analyses, we required that the index sample had not simultaneously had a cytological CIN3+ diagnosis in addition to the AGC diagnosis which left 854 women. For follow‐up analyses of ICC, there were 872 women with AGC but no cytological findings indicative of ICC in the index sample.

**FIGURE 2:**
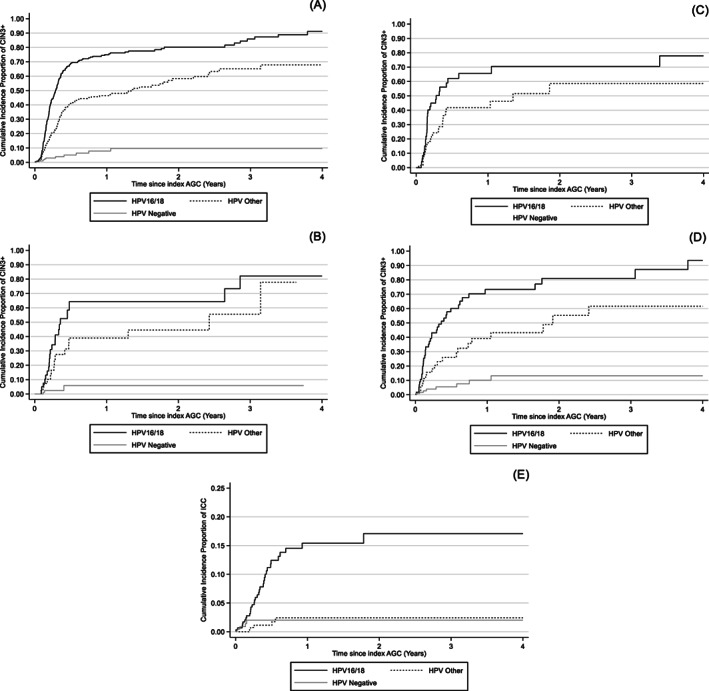
1‐Kaplan‐Meier function on cumulative incidence proportion of CIN3+ or ICC by HPV status. Cumulative incidence proportion of CIN3+ by HPV status among all women with AGC in the cohort (A); among women with AGC who participated to RHS study during 2014 to 2015, randomized to primary cytology (B) and primary HPV analysis (C); among women with AGC diagnosis within the clinically indicated category (D). CIN3+: cervical intraepithelial neoplasia grade 3 or worse including adenocarcinoma in situ. (E) Cumulative incidence proportion ICC by HPV status of an index smear among women with AGC diagnosis ICC: Invasive cervical cancer

HPV16/18 positive women had the highest cumulative incidence proportion of CIN3+ (Table 1) with more than half of these women diagnosed with CIN3+ within the first year of follow‐up (Figure 2A), with similar high risks seen both for primary HPV screening, primary cytology screening and clinical testing (Figure 2B‐D and Table 2). As the use of cytology‐based or HPV‐based screening had been different by calendar time and age, it was particularly valuable that a large proportion of the cohort had been randomized to either cytology‐based or HPV‐based screening, thus avoiding possible sources of bias by factors related to choice of primary analysis. Figure 2B,C compares the cumulative CIN3+ incidence proportions over time in the randomized part of the cohort. The proportions for HPV16/18 and “other HPV” positive AGC appeared to be similar regardless of the primary analysis and the major difference is that the group of HPV‐negative AGC is missing when screening is done with HPV first (Table 2).

**TABLE 1:** Cumulative incidence proportion of CIN3+ and ICC by HPV status of the index sample

HPV status	Outcome	Years after index sample with AGC	At risk at end of follow‐up	Number of cases	Cumulative Incidence proportion (%)	95% CI
HPV16/18	CIN3+	2	19	199	80	74% to 86%
HPV other	CIN3+	2	33	133	58	50% to 66%
HPV negative	CIN3+	2	17	11	10	5% to 18%
HPV16/18	CIN3+	4	4	203	91	84% to 96%
HPV other	CIN3+	4	4	134	68	58% to 77%
HPV negative	CIN3+	4	6	11	10	5% to 18%
HPV16/18	ICC	2	39	33	17	12% to 24%
HPV other	ICC	2	96	5	2	1% to 6%
HPV negative	ICC	2	71	4	2	1% to 5%

**TABLE 2:** The 2‐year cumulative incidence risk of CIN3+ by HPV status of index sample among women randomized to either primary cytology or primary HPV and women with a cervical sample taken by clinical indication

HPV status	Index sample sampling indication	Number of women at risk at end of follow‐up	Survivor function (%)	95% CI
HPV16/18	Primary cytology	4	64	45% to 83%
	Primary HPV	1	70	54% to 85%
	clinical indication	3	81	67% to 92%
HPV other	Primary cytology	7	45	28% to 65%
	Primary HPV	6	59	40% to 78%
	clinical indication	7	55	37% to 75%
HPV negative	Primary cytology	6	6	2% to 20%
	clinical indication	8	13	6% to 27%

The cumulative risk for CIN3+ among the samples taken in clinical testing appeared to have a slightly higher CIN3+ risk after an HPV‐negative AGC (Figure 2D) in line with the fact that there should have been a clinical reason to take the sample.

The proportion of normal or mild/moderate histopathologies seen after HPV‐negative AGC was >95% (230/241 women) and there were about twice as high proportions of normal/mild/moderate histopathologies after an AGC with “other HPV” (203/344 women) than after an AGC positive for HPV16/18 (83/300 women).

The risk to develop invasive cervical cancer (ICC) differed strongly by the HPV status of the index AGC sample (Figure 2E). For HPV16/18 positive AGC the 2‐year cumulative incidence was 17%, whereas both “other HPV” and HPV‐negative AGC had a 2‐year cumulative incidence of ICC of 2% (Table 1). As a very large number of subjects in the cohort developed the outcome, the number of noncases under follow‐up after 2 and 4 years was limited (Table 1).

The Hazard ratios were compared by age group, but no statistically significant differences were seen (Table 3). The Hazard ratios could not be adjusted by indication for taking the sample, as primary HPV screening had no HPV‐negative observations. However, an inspection of the Kaplan‐Meier graphs by indication for taking the sample found only minor differences (Figure 2B‐D).

**TABLE 3:** Hazard ratios for CIN3+ by primary analysis of index sample and frequency of CIN3+ by HPV types and age categories stratified by primary analysis (n = 854)

	Primary cytology		Primary HPV		Clinically indicated	
	Unadjusted	CIN3+/total	Unadjusted	CIN3+/total	Unadjusted	CIN3+/total
	HR (95 %CI)	n (%)	HR (95 %CI)	n (%)	HR (95 %CI)	n (%)
HPV types^a^						
HPV16/18	3.74 (2.62‐5.35)	82 (72.57)	2.17 (1.57‐3.00)	81 (69.83)	5.17 (3.19‐8.40)	40 (70.18)
HPV other	0.95 (0.66‐1.37)	45 (44.12)	0.46 (0.33‐0.63)	69 (38.76)	1.11 (0.66‐1.87)	20 (35.09)
Age						
23–29	Reference	89 (62.24)	0.65 (0.26‐1.65)	5 (45.45)	Reference	17 (36.96)
30–34	0.45 (0.26‐0.79)	15 (34.88)	Reference	49 (63.64)	1.83 (0.93‐3.60)	17 (53.12)
35‐39	0.64 (0.31‐1.33)	8 (30.77)	0.88 (0.57‐1.36)	35 (59.32)	0.90 (0.43‐1.90)	12 (26.67)
40‐44	0.38 (0.17‐0.82)	7 (21.21)	1.00 (0.63‐1.59)	29 (54.72)	1.05 (0.48‐2.30)	10 (37.04)
45‐49	0.33 (0.16‐0.68)	8 (17.78)	0.61 (0.34‐1.08)	15 (39.47)	0.56 (0.19‐1.67)	4 (15.38)
50‐54	0.13 (0.02‐1.00)	1 (6.67)	0.42 (0.21‐0.86)	9 (32.14)	0.26 (0.06‐1.14)	2 (8.33)
55‐59	0.13 (0.02‐0.94)	1 (7.69)	0.22 (0.07‐0.70)	3 (17.65)	0.76 (0.22‐2.60)	3 (16.67)
60+	0.76 (0.10‐5.49)	1 (25.00)	0.60 (0.24‐1.53)	5 (45.45)	0.61 (0.18‐2.11)	3 (15.00)

^a^ Reference category for HPV types was negative for the respective type(s).

We also recorded whether the women with AGC had developed an endometrial cancer. Fourteen women had had an endometrial cancer on follow‐up. Thirteen of these women had had an HPV‐negative AGC.

Finally, we compared whether the proportion of detected invasive cancers differed by primary HPV or primary cytology. As described above, almost 400 000 women were randomized to either primary HPV screening or primary cytology screening. There were four invasive cancers after AGC in the primary cytology arm and five invasive cancers after AGC in the primary HPV arm (no significant difference). The highest proportion of ICC was detected among the 169 women with AGC after primary HPV screening was introduced for all women (15 cases of ICC).

## 4 DISCUSSION

### 4.1 Statement of main findings

We find that HPV16/18 positive AGC has an extremely high risk for CIN3/AIS in subsequent histopathology and, even more striking, for cervical cancer. In contrast, a majority (54%) of the AGC detected by cytology in the most comparable group (organized, randomized to primary cytology; Figure 1) was HPV‐negative and had only small risks, implying that the switch to primary HPV screening has resulted in that the AGC that is now detected has greater risk than the AGC that used to be detected in cytology‐based screening.

### 4.2 Strengths and limitations

Strengths of the study is that it is a very large cohort study, that it encompasses all women with AGC in the population and that reflex HPV testing after AGC had been introduced for all cervical cytologies in the region. Furthermore, there was comprehensive, population‐based registry‐based follow‐up in national registries resulting in that all cases of high grade or invasive cervical disease was captured even if women had relocated to other areas of Sweden. Also, the fact that a large part of the cohort had participated in a randomized healthcare study that randomized into HPV screening or cytology screening enabled an assessment of whether the progressive potential of the AGC differed if the HPV test or the cytology had been performed first, in a manner free from biases related to the reason why HPV or cytology testing had been chosen. Finally, the management guidelines for AGC had been kept identical and independent of the HPV test result, resulting in that ascertainment bias by differential intensity in assessment is unlikely.

Limitations of the study include that the HPV testing was not randomized but performed on all women with AGC and that the reason why a physician had ordered a clinically indicated cervical test was not recorded. Also, the recommended routine to follow‐up an AGC with colposcopy‐directed biopsy was not always followed: only 84% of the women with AGC actually had a cervical biopsy taken.

### 4.3 Comparison to others

Verdoodt et al^15^ performed a systematic review and meta‐analysis of AGC. Only 12 studies were identified. The average proportion of AGC that was HPV positive was 40%. Patadji et al^16^ performed a large retrospective study (n = 3709) on AGC and Hybrid Capture 2 HPV testing in USA. They found that only 28% of the AGC were HPV positive, much less than in our study. The high proportion of HPV negativity in AGC contrasts to the regular presence of HPV in squamous precursor lesions. For example, already 10 years ago a systematic review found about 5000 HPV‐tested cases of CIN2, of which only 15% were HPV negative and among 12 000 cases of CIN3 only 8% were HPV negative.^17^

Several recent studies have further strengthened the evidence indicating that HPV is a strong progression marker among women with AGC.^18^, ^19^

We have previously reported a follow‐up that was restricted to primary cytology screening^20^ and reported a similar proportion of HPV‐positive AGC (56%). The major difference compared with our older study was that the proportion of women with AGC that had actually been followed up had increased (84% in the present study, compared with 70% in the older study). A likely explanation is that following our previous studies quantifying the cervical cancer risk after AGC^5^ there has been increased awareness of the fact that AGC are actually high‐risk precursors of cervical cancer.

### 4.4 Implications

The very high risk for invasive cervical cancer after an HPV16/18‐positive AGC, implies that an ambitious clinical assessment is required, such as colposcopy at an expert referral center. The intermediate risk after “other HPV” positive AGC implies that close follow‐up is warranted. The low risk after an HPV‐negative AGC implies that the management of this group could be less intense and that the recent switch to HPV‐based screening (which means that HPV‐negative AGC will no longer be detected) does not result in any substantial loss in diagnostic accuracy. Rather, it has been proposed that after the introduction of liquid‐based cytology and HPV screening, AGC may have become easier to detect.^21^, ^22^

Primary HPV screening has been shown to be superior in protection against invasive cervical cancer,^10^ with the improved protection seen almost exclusively for improved protection of cervical adenocarcinoma.^10^ The results of the present study suggest that improved detection of adenocarcinoma precursor lesions may be easier if the high‐risk lesions (HPV16/18‐positive AGC) is not mixed with low risk‐lesions (HPV‐negative AGC) and that further improvements in the effectiveness of HPV‐based screening may be obtained if detection and management of HPV16/18‐positive AGC is further optimized.

## AUTHOR CONTRIBUTIONS

The study reported in the paper has been performed by the authors, unless clearly specified in the text. Ingrid Norman: data curation, lead, investigation, writing—review & editing. Klara Miriam Elfström: data curation, supporting, supervision, supporting, formal analyses, supporting, writing—review & editing. Maria Hortlund: formal analyses, supporting, writing—review & editing. Anders Hjerpe: supervision. Joakim Dillner: conceptualization, funding acquisition, project administration, resources, supervision, lead, validation, writing—original draft, writing—review & editing. Emel Yilmaz: formal analyses, lead, visualization, writing—review & editing. All authors have approved the final version for submission.

## FUNDING INFORMATION

This study was supported by the Swedish Association of Local Authorities and Regions for the Swedish National Cervical Screening Registry; and by the Swedish Cancer Society (18 0443 and 20 1198 PjF to Joakim Dillner).

## CONFLICT OF INTEREST

The authors declare no conflict of interest.

## ETHICS STATEMENT

The study was approved by the ethical review board (ERB) of the Stockholm Region (Decision number 16/1103‐31). The ERB decided that consent was not required and thus all women resident in the population of the region could be included in the study.

## Data Availability

The data underlying this article can be requested from the corresponding author.
